# Percussion test: description and diagnostic accuracy of a new manual test for bone marrow edema of the knee

**DOI:** 10.1186/s12891-022-05028-y

**Published:** 2022-01-18

**Authors:** Valerio Sansone, Alessandro Galluzzo, Emanuele Maiorano, Marina Benedetta Polatti, Valerio Pascale

**Affiliations:** 1 Department of Orthopaedics, University of Milan, Milan, Italy; 2 I.R.C.C.S. Istituto Ortopedico Galeazzi, Milan, Italy; 3 Residency Program in Orthopaedics and Traumatology, University of Milan, Milan, Italy; 4 University of Milan, Milan, Italy

**Keywords:** Bone edema, Bone marrow lesion, Diagnostic accuracy, Knee pain, Physical examination, Knee, Prospective observational study, Sensitivity, specificity

## Abstract

**Background:**

Prompt diagnosis of bone marrow lesion (BML) is difficult but critical for correct treatment. Magnetic resonance imaging is the gold standard, although expensive and time consuming. Simple and reliable clinical test for BML detection is lacking. Aim of the study is to describe a new manual clinical test called Percussion Test (PT) and to statistically determine its diagnostic accuracy in BML, compared to MRI imaging.

**Methods:**

After evaluation of the inclusion and exclusion criteria, 218 consecutive patients with unilateral knee pain and age comprised between 18 and 80 years old were enrolled in our observational prospective study. Informed consent was obtained for each patient. After medical history collection, PT was performed by a single operator as described. MRI was performed in the affected knee to detect the presence of BML. Coherence in PT and MRI assessment was evaluated in each quadrant of the knee via contingency tables, as sensitivity, specificity, NPV, PPV and diagnostic accuracy were calculated.

**Results:**

No correlation with a positive PT was demonstrated for the covariables gender (*p* = 0.156), age (*p* = 0.272) and BMI (*p* = 0.639).

PT showed a sensitivity ranging from 60.6 (40.6–80.6) to 79.5 (63.0–96.0) and a specificity ranging from 85.7 (80.0–91.5) to 96.0 (93.1–98.9) depending on knee quadrant. Diagnostic accuracy ranged from 81.6 (75.9–86.6) to 89.4 (84.6–93.2), and *p*-value was < 0.00001 in a chi-squared analysis for all quadrants.

**Conclusions:**

PT showed sensitivity and specificity values that are comparable with other clinical tests routinely adopted in clinical practice. In the absence of other reliable clinical test, PT has the potential to become a useful bedside tool in the diagnosis and management of BMLs.

**Supplementary Information:**

The online version contains supplementary material available at 10.1186/s12891-022-05028-y.

## Background

Over the last recent years, few topics have received as much attention from the orthopedic community as bone marrow edema (BME), at least judging by the remarkably increasing amount of scientific publications on this issue.

Actually, BME is an interdisciplinary topic ranging from orthopedics to traumatology, radiology, rheumatology, physical medicine, up to internal medicine, endocrinology, diabetology, oncology, pediatrics, and beyond.

Compared to the first descriptions reported in the literature at the end of the ‘80 [1], a significant amount of data has been achieved, leading to a better comprehension of the mechanisms underlying the disease, although several aspects remain to be clarified. As evidence of this progress, at least three classifications [2–4] have been proposed and updated over the years. Moreover, the definition of the condition has also been modified, introducing the more appropriate term of Bone Marrow Lesion (BML) [5, 6].

However, while significant progress has been made in understanding pathophysiology and treatment, the same cannot be affirmed for the diagnosis, which continues to rely primarily on MRI (Magnetic Resonance Imaging). However, the limitations of MRI are well known: the cost of the examination, the relatively not ubiquitous distribution throughout the territory, and the length, sometimes very long, of the waiting lists. As a matter of fact, also because of the lack of symptom specificity, the correct diagnosis often is delayed and the condition may be misdiagnosed and treated incorrectly, if not untreated [7, 8].

Yet, prompt identification of BML would be crucial because its presence, beyond the disabling pain, is a well-known negative prognostic factor of progression of knee osteoarthritis with increased structural deterioration, cartilage damage, and reduced function [9–13].

Furthermore, some authors support the hypothesis that BML may represent an early stage of avascular necrosis [14, 15].

Thus, given the disabling symptoms of bone edema and its potential unfavorable evolution, a simple and reliable method to ascertain BMLs presence and to monitor their course would be highly desirable.

Therefore, we sought to develop a clinical test that would be easy to perform but, at the same time, would have sufficient reliability in diagnosing knee BMLs. Our test is based on the common clinical observation that bony areas affected by BMLs are painful on digital pressure. The explanation for this phenomenon could be reasonably due to the sudden rise of intramedullary pressure into the bone compartment due to the external percussive force. We named it “Percussion Test” (PT) and it is characterized by the manual percussion of the bony prominence of the knee to be evaluated. The test, in a way, takes inspiration from the classical semeiotics of the past, when instrumental diagnostics was not as developed as it is today, nor easily available. To date, no clinical test for diagnosing bone edema has been described, with the unique exception of the “tapping test” reported by Aigner et al. [16] However, performing this test requires the use of an apparatus consisting of a reflection hammer hinged to a support bracket, whose purpose would be to provide a reproducible percussive force. In brief, it is not exactly a simple test available in any clinical setting.

The aim of the present study was to evaluate the diagnostic accuracy of a manual test in detecting knee BMLs. The only tool needed is the examiner’s bare hands.

## Materials and methods

This was a single center observational prospective study in the context of a large academic hospital. In an ambulatory setting, initially, every patient underwent accurate anamnestic collection about past medical history and history of present illness. All patients referring knee issues was evaluated for study enrollment. Inclusion criteria were presence of unilateral knee pain and age comprised between 18 and 80 years. Exclusion criteria were presence of recent wounds around the knee and inflammatory processes involving overlying skin, including ecchymosis and hematoma. From October 2018 to October 2019, 218 patients were included in the study, 99 males and 119 females. Demographic data were collected. At the time of clinical assessment, the mean age was 55 ± 13,6 years, and mean BMI was 25,9 **±** 4,8 kg/m^2^. The clinical assessment was carried out by the same investigator during his usual clinical practice. Along with all other procedures, the investigator performed PT for detecting BML. PT was performed with the patient sitting and the calves hanging; the manual percussion was performed with two fingers and was targeted to the medial and lateral femoral condyle, and to the medial and lateral tibial plateau. The percussion of the femur was performed on the medial aspect of the medial condyle and on the lateral aspect of the lateral condyle, whereas the percussion of the tibia is performed on its anterior aspect, medially to the anterior tibial tuberosity for the medial plateau and just anterior to the fibular head for the lateral one. The test was always performed bilaterally, in order to define its positivity as the asymmetry of the pain perceived between the painful knee and the healthy one. In presence of a similar finding on both knees, the outcome was considered negative.

Subsequently, maximally 6 weeks after the clinical assessment, all patients underwent a knee MRI as the gold standard for detecting BML presence. Coronal, axial and sagittal T1-weighted sequences were performed, followed by coronal, axial and sagittal T2-weighted sequences and T2-weighted fat-suppressed sequences using a 1.5 T MRI scanner. The frontal planes of the T2-weighted images were divided into 4 quadrants, in order to define the exact location of BML [16]. Clinical evaluation data and MRI findings were collected in a database as presence or absence of BML. All subjects signed an informed consent form and the study was approved by a local ethics committee. The study was performed according to the Declaration of Helsinki principles for medical research involving human subjects.

Data derived from the database were independently analyzed for each quadrant of the knee. Age and BMI were reported as mean standard deviation. The sensitivity, specificity, positive, and negative predictive values of the test were calculated for each quadrant, as well as diagnostic accuracy. CI95% was reported. Chi-squared analysis of contingency tables was performed. A regression analysis was also performed to verify if the covariates age, gender, and BMI influenced the clinical test response. All statistical analyses were conducted with the IBM SPSS Statistics 24 software program (IBM Corporation, Armonk, NY), and the critical value for significance was set at *P* <  0.05.

## Results

218 patients were included in the study. 73 patients reported traumatic onset of pain (knee sprain, direct trauma), 38 patient reported acute (under 6 weeks) non-traumatic onset of pain while 107 patients reported chronic (over 6 weeks) non traumatic onset of pain. 79 patients showed a BML on MR imaging in 0 quadrants, 78 patients showed BML in 1 quadrant, 56 patients showed BML in 2 quadrants, 4 patients showed BML in 3 quadrants, 1 patient showed BML in all 4 quadrants of the knee. BML was located in the medial femoral condyle in 53 cases, the medial tibial plateau in 78, the lateral femoral condyle in 33, and the lateral tibial plateau in 42 cases. The observed overall BME prevalence in this population sample is 64.2%, this observation being consistent with literature [17].

Contingency tables for each quadrant are reported in Table 1. Sensitivity, specificity, positive, and negative predictive values are reported in Table 2. Chi-squared analysis of contingency tables is reported in Table 3.

**Table 1:** Contingency tables for each knee quadrant. PT, percussion test; MRI, magnetic resonance

		MRI positive	MRI negative
Medial femoral condyle	PT positive	34	21
	PT negative	19	144
Lateral femoral condyle	PT positive	20	10
	PT negative	13	175
Medial tibial plateau	PT positive	62	20
	PT negative	16	120
Lateral tibial plateau	PT positive	26	7
	PT negative	17	168

**Table 2:** Sensitivity, specificity, positive, and negative predictive value, and accuracy values for each knee quadrant. The values are expressed in percentage (CI95% in brackets)

	Sensitivity	Specificity	PPV	NPV	Accuracy
Medial femoral condyle	64.2 (44.6–83.7)	87.3 (82.2–92.4)	61.8 (49.0–74.7)	88.3 (83.4–93.3)	81.6 (75.9–86.6)
Lateral femoral condyle	60.6 (40.6–80.6)	94.6 (91.3–97.9)	66.7 (49.8–83.5)	93.1 (89.8–96.7)	89.4 (84.6–93.2)
Medial tibial plateau	79.5 (63.0–96.0)	85.7 (80.0–91.5)	75.6 (66.3–84.9)	88.2 (82.8–93.7)	83.5 (77.8–88.2)
Lateral tibial plateau	60.5 (40.5–80.4)	96.0 (93.1–98.9)	78.8 (64.9–92.7)	90.8 (86.6–95.0)	89.0 (84.1–92.8)

**Table 3:** Chi-squared analysis of contingency tables. *χ*^2^ is calculated with 1 degree of freedom and *N* = 218. Significance is set at *p* < .05

	*χ*^2^	*p*
Medial femoral condyle	56.23	< 0.00001
Lateral femoral condyle	71.90	< 0.00001
Medial tibial plateau	90.75	< 0.00001
Lateral tibial plateau	85.67	< 0.00001

No correlation with a positive PT was demonstrated for the covariables gender (*p* = 0.156), age (*p* = 0.272) and BMI (*p* = 0.639).

Discussion

PT showed high specificity values (85–96%) and relatively low sensitivity values (60–79%). Sensitivity and specificity values of PT shown in this study are comparable with the diagnostic accuracy of several functional evaluation tests widely adopted in everyday clinical practice. For example, the anterior drawer test for anterior cruciate ligament (ACL) insufficiency has a sensitivity ranging from 25% [18] to 91% [19] and a specificity ranging from 77% [20] to 100% [21] according to the literature. The Neer test, probably the most common routinely in use for the evaluation of shoulder impingement, has a sensitivity ranging from 33% [22] to 89% [23] and a specificity ranging from 30% [24] to 69% [25]. PT sensitivity shown in this study is lower than tapping test sensitivity reported by Aigner et al. [16] but positivity evaluation is conceptually different. They performed the tapping test with a hinged mallet, and the patient reported perceived pain on a visual analogical scale (VAS). VAS-reported pain is highly subjective, and it is very difficult to set a universal threshold to separate positive from negative results. Regarding this aspect, one of the advantages of our PT is the definition of a “positive” result. Patients are asked to compare the effect of the percussion stimulus received on both knees. PT is considered positive when the percussion is perceived differently in the examined knee, being either more painful or simply more pronounced. In this way, the patient performs an immediate internal analysis of the consequences of the mechanical stimulation, allowing the clinician to eliminate the bias deriving from the subjective threshold of pain.

The ideal application for the PT in clinical practice would be to implement it as a screening tool, to help identify a pool of patients with a heightened probability of BML, that are worthy or MRI imaging in terms of cost effectiveness. However, sensitivity is too low for the PT to be adopted as an effective screening tool.

Nevertheless, in the light of the high specificity we have observed, a positive result may be considered reliable. This implies that PT might be used in the early stages of the diagnostic pathway, to anticipate treatment, avoiding delays caused by the, often not immediate, MRI availability.

Moreover, a high negative predictive value of the PT may be employed to reduce the number of MRI controls during the follow-up period. The high negative predictive value (NPV) found (88–93%) suggests that a patient diagnosed with BML but with a PT turned negative at follow-up, has a significant probability of being cured or, at least, to have experienced a significant reduction in BML’s amplitude. Regarding the latter, previous studies have observed a significant correlation between the extent of BMLs and pain [26, 27].

In the absence of new anamnestic and clinical findings suggesting the presence of soft tissue lesions (e.g., meniscal tears), MRI might be avoided as the BML healing process can be monitored with a serial assessment of PT.

The major advantage of the PT lies in its simplicity and cost-effectiveness: it is intuitive, quick, and easy to perform. Moreover, no equipment is required and the learning curve seems to be really steep. Strength of the study is the large number of patient and the consistence of the data extracted, which comes from having all MRIs performed in one centre and the test performed by a single examiner.

A potential weakness lies in its reproducibility, although this is a common issue of every clinical manual test. In awareness of this, we intend to assess the inter and intra-observer variability in future studies.

## Conclusion

In this retrospective observational study, the newborn PT showed moderate sensitivity (60–79%) and good specificity (87–96%). The most attractive aspects of the PT are its handiness and ease of execution since it can be replicated in every clinical context and no special tool is required. Overall, PT might have the right features to become a useful bedside tool in the diagnosis and management of BML.

## Supplementary Information

**Additional file 1.**

## Data Availability

All data generated or analysed during this study are included in the supplementary information files.

## Abbreviations

BME: Bone Marrow Edema
BML: Bone Marrow Lesion
MRI: Magnetic Resonance Imaging
PT: Percussion Test
ACL: Anterior Cruciate Ligament
VAS: Visual Analogical Scale
NPV: Negative Predictive Value
CI95%: Confidence Interval 95%
